# Integrating Metabolic and MicroRNA Profiling to the Diagnostics of Endometriosis: A Pilot Study

**DOI:** 10.3390/ijms27073052

**Published:** 2026-03-27

**Authors:** Yaroslav D. Shansky, Sulejman S. Esiev, Uliana V. Pokazannikova, Yulia V. Kudryavtseva, Lyudmila A. Chursina, Julia A. Bespyatykh

**Affiliations:** 1 Department of Molecular Medicine, Center of Molecular Medicine and Diagnostics, Yu.M. Lopukhin Federal Research and Clinical Center of Physical-Chemical Medicine of Federal Medical Biological Agency, Malaya Pirogovskaya Str., 1a, 119435 Moscow, Russia; kap20081@gmail.com (S.S.E.); julia_kudriavtseva@mail.ru (Y.V.K.); juliabes@rcpcm.org (J.A.B.); 2 Department of Gynecology, Clinical Hospital No. 123, Federal Research and Clinical Center of Physical-Chemical Medicine of Federal Medical Biological Agency, Malaya Pirogovskaya Str., 1a, 119435 Moscow, Russia; ulyana.pokazannikova@yandex.ru (U.V.P.); ludachursina@yandex.ru (L.A.C.); 3 N.V. Sklifosovskiy Institute of Clinical Medicine, I.M. Sechenov First Moscow State Medical University, Ministry of Health of the Russian Federation, Trubetskaya Str., 8, b. 2, 119048 Moscow, Russia; 4 Department of Expertise in Doping and Drug Control, Mendeleev University of Chemical Technology of Russia, Miusskaya Square, 9, 125047 Moscow, Russia

**Keywords:** polyunsaturated fatty acids, miRNA, gas chromatography, mass-spectrometry, multivariate analysis, metabolic profiling

## Abstract

Endometriosis affects a large number of women of reproductive age, and its pathogenesis is still unclear. It causes severe chronic pelvic pain, which is often misdiagnosed as irritable bowel syndrome, or other disorders. Metabolomics and transcriptomic approaches enable the study of changes in various physiological or pathological pathways to identify new potential biomarkers. We employed gas chromatography–mass spectrometry (GC–MS) to investigate metabolic alterations, and quantitative real-time polymers-chain reaction (RT-qPCR) to assess changes in miR-451a and miR-125b in saliva in endometriosis. Serum and saliva samples of patients with symptomatic endometriosis and volunteers without it were collected and subjected to GC–MS and qPCR-RT analysis, respectively. Multivariate and univariate statistical analyses were performed. Orthogonal partial least squares discriminant analysis has shown the differences between the two groups. Eicosadienoic acid, arachidonic acid, and miR-451a increased significantly in endometriosis patients. Machine learning methods were used to build the predictive model, which can be used in early low-invasive diagnostics of endometriosis. Receiver operating characteristics analysis has tested the diagnostic power of metabolites. The combination of metabolic and microRNA profiling may improve our knowledge of the pathophysiological and signaling mechanisms in endometriosis and the discovery of new efficient biomarkers of endometriosis.

## 1. Introduction

One of the most common chronic diseases of the female reproductive system is endometriosis. The most common manifestations of endometriosis are chronic pelvic pain syndrome, menstrual irregularity, and infertility [1,2,3]. Along with these symptoms, endometriosis significantly reduces a woman’s quality of life, and secondary infertility (~30% to 50% of cases) creates a major social challenge [4,5,6]. Additionally, endometriosis carries an economic burden. The annual costs of endometriosis treatment in Europe are similar to those of other chronic diseases, such as diabetes [7].

A common method used in clinical practice to diagnose endometriosis is transvaginal ultrasound of the pelvic organs. However, to confirm the diagnosis, histological examination data are required alongside laparoscopy and direct visualization of endometriosis lesions. Aside from the initial non-specific symptoms of the disease, it involves invasive diagnostic procedures. When combined, these factors contribute to a delayed diagnosis. Serological protein markers (CA125, CA 15-3) lack specificity and have limited diagnostic significance, primarily in stage III-IV endometriosis [8,9,10].

Since a single biomarker has limited information, it is important to consider a set of substances that can distinguish one patient condition from another. Additionally, endometriotic lesion development occurs via multiple pathways [11], and various molecules influence the phenotypic differences of the disease [12]. Recent years have seen the use of omics approaches to explore the mechanisms of endometriosis and develop diagnostic methods. Genetics offer extensive data, especially through next-generation sequencing and genome-wide association studies [13], but the invasive sampling and high costs limit the use of these genomic methods in clinical practice [14]. Proteomics take a closer look at proteins that directly mediate cellular functions and disease mechanisms. Proteins’ expression levels are influenced less by external factors like diet or medication, and this approach maintains high sensitivity, specificity, and throughput. It can reveal potential markers, although the high costs, difficulty in detecting low-abundance proteins, and lack of clinical validation remain its disadvantages [14,15]. Metabolomics, the newest omics field, analyzes various biological samples with high sensitivity and specificity, assessing how external influences impact metabolic pathways. Changes in the metabolome linked to endometriosis have been identified in numerous studies [14,16,17], creating opportunities to discover new, minimally invasive diagnostic markers for the disease [18,19]. Over the past 15 years, the number of publications on “endometriosis [MeSH Terms] AND (metabolomics [MeSH Terms] OR metabolome [MeSH Terms])” has increased roughly tenfold, according to PubMed. One of the most promising diagnostic approaches is metabolomic analysis using mass spectrometry (MS). Studies employing this technique have made it possible to identify potential biomarkers for numerous diseases, including endometriosis. One of the most promising approaches for diagnostics is metabolomic analysis performed by mass spectrometry (MS) methods. Studies conducted using this approach enable the identification of potential biomarkers for various diseases, including endometriosis [20,21,22,23].

Recent studies have established a significant relationship between the level of polyunsaturated fatty acids (PUFAs) and the development of endometriosis [24,25,26]. The biological roles of PUFAs are diverse and not specific to endometriosis, which makes it challenging to use them as standalone biomarkers. However, evidence suggests that ω-3 PUFAs may reduce inflammation in patients with endometriosis by lowering levels of pro-inflammatory cytokines such as TNF-alpha, IL-6, and IL-1, indicating potential anti-inflammatory properties that deserve further research [27,28]. The interest in fatty acids, especially PUFAs, stems from their well-known role in inflammatory processes. Arachidonic acid (AA) is a key precursor of various eicosanoids, including thromboxanes, prostaglandins, and leukotrienes [25,29]. Prostaglandin E2 (PGE2) increases estrogen synthesis and inhibits apoptosis through fibroblast growth factor-9 (FGF-9), promoting cell proliferation [22]. Meanwhile, thromboxanes enhance thrombocyte aggregation, which can lead to circulation failure in endometrial tissue [21]. Leukotrienes are crucial in initiating chronic inflammation [29]. PUFAs also contribute to membrane lipid synthesis and serve as the primary energy source for cells. The survival of ectopic endometrial cells and their resistance to apoptosis are directly linked to altered lipogenesis [30]. Therefore, PUFAs could serve as potential biochemical markers for endometriosis related to its pathogenesis. The main analytical technique for studying PUFAs is gas chromatography coupled with mass spectrometry (GC–MS), which enables the preliminary separation of substances in the analyzed sample. This method is valuable for assessing PUFAs levels and the omega-3 index, which is associated with conditions such as increased risk of coronary heart disease and low bone mineral density [31]. Analysis and interpretation of data involve methods for developing multiparametric classification models to evaluate the diagnostic accuracy of identified endometriosis biomarkers. Characteristic mass spectra, also called “fingerprints,” are used to create a molecular classification model that assigns serum or plasma samples to normal or pathological groups. However, detailed data on PUFAs profiling by gas chromatography coupled with mass spectrometric detection (GC–MS) in healthy individuals and endometriosis patients are not available.

Metabolomics approaches are challenged by the high diversity of metabolic parameters, which are influenced by individual differences and external inputs. The effectiveness of omics approaches improves when combined with others, such as genomics or proteomics [32]. Transcriptomics and RNA profiling are the promising methods that assess RNA molecule levels in the human body. Among these molecules, microRNA, or miRNA/miR molecules are potential markers of endometriosis [33] and cancer diseases [34,35]. MicroRNA miR-451a, as measured in saliva in our study, can regulate IL-6R and the JAK/STAT/TF signaling pathway, affecting angiogenesis, inflammation, and hypercoagulability [36,37]. Moreover, miR-451a acts as an anti-inflammatory regulator by targeting key signaling nodes such as MIF/PI3K/AKT and transcription factors (ATF2, YWHAZ) [38,39]. It also may regulate PTEN expression, contributing to the excessive growth of endometrial tissue when mediated with other miRNAs like miR-25 [40]. Additionally, miR-451a modulates inflammation and stem-like properties via the IL-6/STAT3 pathway, potentially mediating growth of endometrial lesions and tissue injury.

Other miRNAs, including miR-25, mediate this process, and as a result, miR-451a promotes excessive endometrial tissue growth [40]. One advantage of using miRNAs is their high diagnostic sensitivity in saliva, opening new possibilities for minimally invasive diagnostics of women’s reproductive diseases. The roles of many miRNAs in signaling and regulatory pathways of endometriosis have been demonstrated, and numerous diagnostic signatures (7–10 miRNAs) have been proposed [10,36,37,41].

MicroRNA biomarkers demonstrate high sensitivity and specificity when combined, although their analysis can be costly. However, costs decrease with the development of routine techniques such as quantitative PCR-RT with reverse transcription (qPCR-RT). Commercial RT-qPCR kits enable fast and accurate measurement of miRNA levels in various biological fluids. Finally, miRNA biomarkers show high sensitivity and specificity when combined, offering promising tools for diagnosing women’s reproductive diseases through minimally invasive methods as they are developed [33,36,42,43].

The functional analysis of miRNAs can be performed using various techniques, such as microarrays, northern blotting, real-time polymerase chain reaction, and RNA sequencing. However, the “gold standard” for miRNA analysis is qPCR-RT, because of its high precision, accuracy, and broad range. Many commercial kits are available to measure miRNA levels in biological samples via qPCR-RT [44]. Additionally, this technique is the most cost effective for profiling of a low number of miRNAs.

Therefore, multi-omics techniques have emerged as a promising approach for the diagnosis of endometriosis and its risk assessment. Here, we studied the possibility of distinguishing patients with endometriosis (stages I-IV) from healthy volunteers by analyzing targeted PUFAs in blood samples. This included the simultaneous evaluation of blood PUFAs levels and RNA profiling of saliva samples, followed by statistical analysis of the obtained data. Metabolic profiling of blood serum was performed using GC–MS to measure the levels of four PUFAs: *all-cis*-eicosatetraenoic (arachidonic) acid, AA; *cis*, *cis*-11,14-eicosadienoic acid, EDA; *all-cis*-docosahexaenoic acid, DHA; *all-cis*-eicosapentaenoic acid, EPA) associated with the pathogenesis of endometriosis in 52 serum samples. The profiling of miR-451a and miR-125b was conducted using RT-qPCR on the same set of samples. The results of this prospective study are presented and discussed in the context of non-invasive clinical and laboratory diagnostics.

## 2. Results

### 2.1. Description of the Cohort

The clinical characteristics of the patients in the endometriosis and control groups are presented in Table 1. Among the fifty-two patients, twenty-eight had endometriosis (I-IV stages), and twenty-four were healthy volunteers.

### 2.2. Overview of the Study Pipeline

**Figure 1:**
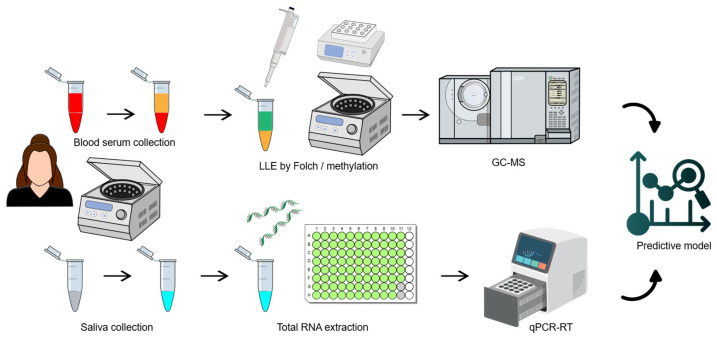
The study pipeline used in this work.

Further analysis employed a multi-omics approach. First, PUFAs were extracted from human blood serum using the modified Folch method, then derivatized with methanol and analyzed by GC–MS to identify FAMEs. The targeted metabolites included AA (HMDB0001043, KEGG C00219), EDA (HMDB0005060, KEGG C16525), EPA (HMDB0001999, KEGG C06428), and DHA (HMDB0002183, KEGG C06429). Simultaneously, miR-451a and miR-125b were isolated from saliva through chloroform/methanol extraction and quantified via RT-qPCR. The molecules showing the greatest differences were used to develop a predictive model using discriminant analysis, multiple regression, and machine learning. This model underwent cross-validation (see Figure 1).

### 2.3. Validation of GC–MS Method

#### 2.3.1. Linearity, LODs, and LOQs

Table 2 summarizes the linearity and range of the calibration curves, including the limit of detection, LOD, and limit of quantitation, LOQ. Linearity has been retained over the concentration range of 2.0–125.0 μg/mL (except for DHA, for which the range was 8–250.0 μg/mL). The linear ranges for these PUFAs were slightly adjusted to cover their serum levels in clinical samples. Sensitivity (i.e., LOD) was calculated as follows (1):

(1) $$\mathrm{LOD}=\frac{3\times \mathrm{SE}(b)}{a}$$

where a is the slope of the linear fit and SE(b) is the standard error of the intercept b. LOQ was calculated as 3 × LOD. The regression coefficient (*r*^2^) for the calibration curves was higher than 0.99 in all PUFAs. The LODs (S/N = 3) of studied PUFAs ranged from 0.27 to 2.37 μg/mL. The LOQs ranged from 1.0 to 7.5 μg/mL for all PUFAs at S/N ≥ 10.

#### 2.3.2. Selectivity and Carry-Over

The results of selectivity and carry-over estimation are summarized in Table 3. The only peak in the double blank solution corresponded to EDA, but it was <15.0% of that one in the LLOQ solution, whereas no peak was observed for any other PUFAs. These results show that the analysis method is selective. Furthermore, there was no significant carryover (<15.0%) for all PUFAs; they ranged from 0.0% (HEA) to 14.8% (EDA) of the peak area at LLOQ.

#### 2.3.3. Within-Run and Between-Run Accuracy and Precision

The accuracy and precision of the within- and between-run assays were evaluated using five replicates of QC samples at four levels: LOQ, 3 × LOQ, 62.5, and 125.0 μg/mL for EDA, AA, and EPA; and LOQ, 3 × LOQ, 187.5, and 250.0 μg/mL for DHA. The intra- and inter-day results for EDA, AA, EPA, and DHA are summarized in Table 4. The within-run accuracy ranged from 86.5% to 107.4%, while the inter-day accuracy ranged from 87.7% to 108.4% across all tested concentration levels for all PUFAs. Additionally, the between-run precision varied from 2.6% to 13.9%, and the inter-day precision ranged from 1.8% to 12.3% for all PUFA concentration levels.

Thus, the GC–MS method was successfully validated in accuracy, linearity, carry-over, sensitivity, and specificity parameters.

### 2.4. The Plolyunsaturated Fatty Acids Profile of Blood Serum

**Figure 2:**
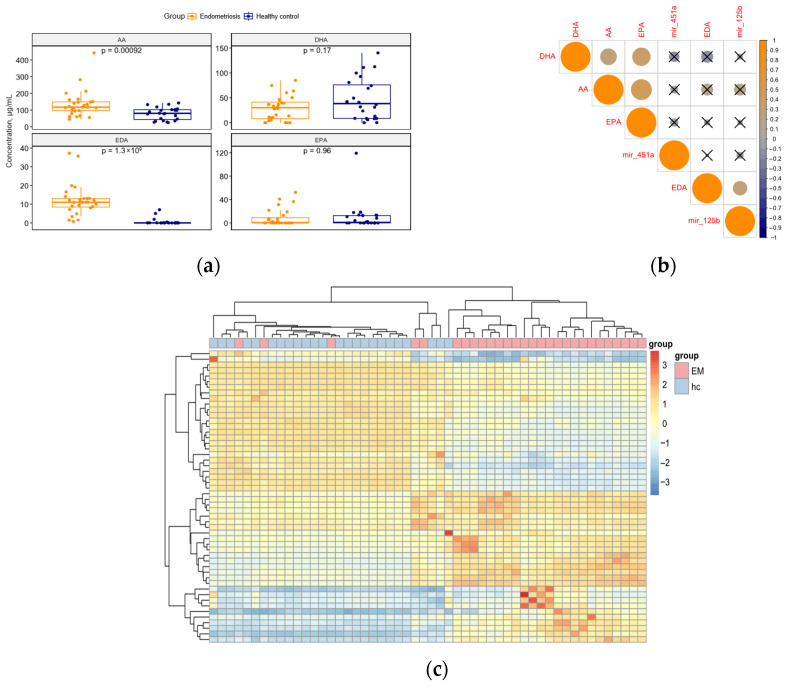
(**a**) The comparison of PUFAs levels in the endometriosis cohort and in the healthy volunteers’ group. (**b**) Correlation matrix for PUFAs and miR-125b, -451a. Crosses indicate that Spearman’s coefficient of correlation does not significantly differ from 0 (*p* > 0.05); otherwise, this difference is significant (*p* < 0.05). (**c**) Heatmap of total ion chromatograms of methylated lipid extracts in endometriosis patients and healthy volunteers (Z-values for Pearson’s coefficients of inter-individual correlation are presented). Despite some outliers, the clusters corresponding two experimental groups can be seen.

The levels of PUFAs (AA, EDA) differ markedly between patients with and without endometriosis: in those with endometriosis, the amounts of AA and EDA in blood serum significantly increase (*p* < 0.002, *p* < 0.001, respectively). No significant differences were observed between the groups in the levels of EPA and docosahexaenoic acid (DHA; *p* = 0.960, 0.160, respectively) (Figure 2a).

The levels of AA, DHA, and EPA are significantly correlated with each other (*p* < 0.05), with correlation coefficients of 0.30 for AA and DHA, 0.48 for AA and EPA, and 0.37 for EPA and DHA (Figure 2b).

Alongside targeted analysis of FAMEs, the untargeted metabolome was also assessed using the same pretreatment method. Visualization of TIC data (Figure S1) with heat maps shows some variability across groups. It is evident that the EM group mainly clustered together, though some individual samples are dispersed among samples from healthy volunteers (Figure 2c).

There was no significant correlation between PUFAs levels and the day of menstrual cycle in experimental groups. The values of Spearman’s coefficient of correlation were −0.14 (*p =* 0.578), −0.08 (*p =* 0.570), −0.16 (*p* = 0.602), and −0.14 (*p* = 0.775) for AA, EDA, EPA, and DHA, respectively.

### 2.5. The Levels of miR-125b and miR-451a Differ in Saliva

**Figure 3:**
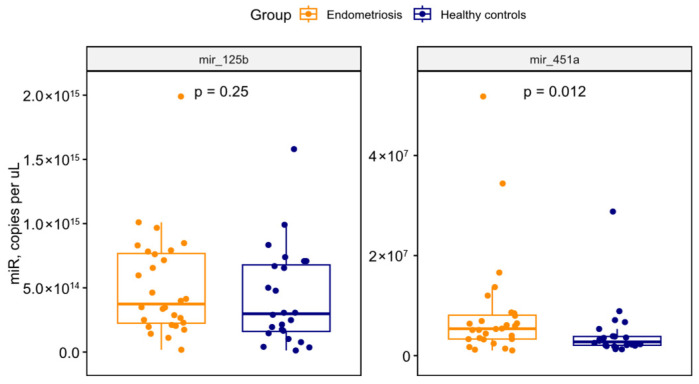
Univariate comparisons between endometriosis patients and healthy volunteers’ samples. Boxplots are presented by Mann–Whitney test.

The miR-451a level was significantly higher in endometriosis patients than in healthy volunteers (Figure 3). Meanwhile, the miR-125b level showed no significant difference between the groups.

There was no significant relationship between miRNA levels and menstrual cycle day in either healthy controls or endometriosis patients. Specifically, for miR-451a, Spearman’s correlation coefficients were −0.11 (*p* = 0.620) in the endometriosis group and −0.033 (*p* = 0.880) in healthy volunteers. For miR-125b, the coefficients were 0.32 (*p* = 0.130) and −0.19 (*p* = 0.390) in the same respective groups.

Interestingly, miR-125b levels showed a significant positive correlation with blood serum EDA concentration, with a Spearman’s coefficient of 0.22 (*p* = 0.049, see Figure 2b).

### 2.6. Predictive Model of Endometriosis

Furthermore, four ML methods were used to build a predictive model for endometriosis: OPLS-DA, a binary logistic model, SVM, and random forest.

#### 2.6.1. OPLS-DA Results

**Figure 4:**
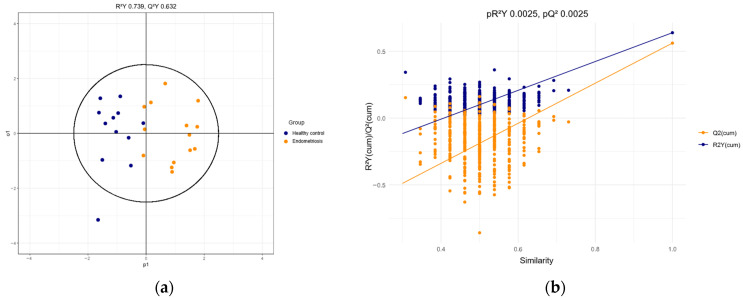
Multivariate analysis of serum samples. (**a**) Model OPLS-DA score plot for endometriosis and healthy control group (with 50% subset as training sample). (**b**) Validation of the model via permutation test (*n* = 400).

The OPLS-DA model was initially trained on a 50% subset of the total samples. This split created two halves with similar sample proportions across each class (see Table A1). The results for the combined data on PUFAs (AA, EDA, DHA, EPA) and miRNAs (miR-451a, miR-125b) show significant prognostic value in distinguishing test samples from patients with and without endometriosis. The model fits the data well (R^2^Y 0.739), effectively differentiates between the two groups, and demonstrates strong predictive power (Q^2^Y 0.672) (see Figure 4a). Its accuracy on the test set was 80.9% (Table 5). 

To validate the model, a permutation test with 400 iterations was performed. The R^2^ intercept was −0.43, and the Q^2^ intercept was −0.94 (*p* = 0.0025) (see Figure 4b).

The independent parameters for logistic classification models were selected based on either a VIP score > 1 or a significant Mann–Whitney U test (*p* < 0.05). VIP ≥ 1 was noted for EDA and AA (with values of 1.877 and 1.075, respectively), and salivary miR-451a levels were higher in endometriosis patients than in healthy controls (*p* = 0.010).

#### 2.6.2. Logistic Binary Regression Results

The equations of logistic binary regression model for differential diagnosis of endometriosis vs. healthy volunteers, is (1):

(2) $$P=\frac{1}{1+e^{\left(-1.405+1.130\times EDA+0.008\times AA+2.808\times 10^{-7}\times MIR\_451a\right)}}$$

The impact of DHA, EPA, and miR-125b to regression model was non-significant.

The ROC-analysis has shown the perfect predictive ability of the model. Its parameters are provided in Table 6.

**Figure 5:**
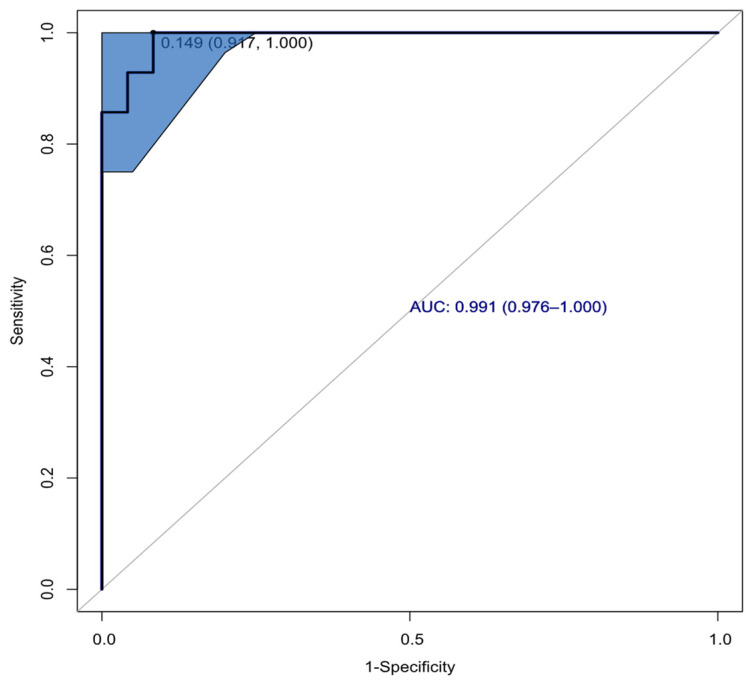
Receiver operating characteristic curve (blue line) obtained after cross-validation for logistic regression model developed (1). The AUC (CI AUC, light-blue area on graph) and cut-off (sensitivity, specificity) values are presented in the graph.

AUC was 0.994; sensitivity and specificity were 0.958 and 0.964 in cut point with corresponding accuracy 0.962 (Figure 5).

#### 2.6.3. SVM Results

The linear SVM kernel method was further applied to the data to identify its discriminative features. The dataset was divided into a training set (80%) and a test set (20%). The process ran for 50 iterations.

The model achieved an accuracy of 0.962 when using EDA, AA, and miR-451a. Adding EPA, DHA, and miR-125b improved accuracy slightly to 0.981 (see Table 5).

#### 2.6.4. Random Forest Results

**Figure 6:**
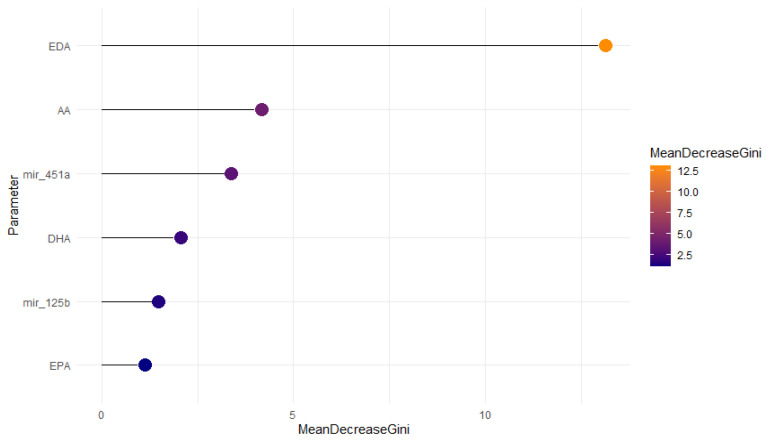
Variable importance plot for the predictive model. EDA, AA, and miR-451a show the largest impact to the predictive ability.

The random forest method was also used to identify their key discriminative features. The dataset was divided into a training set (75%) and a test set (25%), with 100 iterations. The model performed well, with an error rate of 9.62% (see Table 5). The most influential variables identified were EDA, AA, and miR-451a, each showing a decrease in accuracy of >1 (36.07, 9.43, and 4.31, respectively). The average decrease in the Gini index for these variables is shown in Figure 6.

## 3. Discussion

Endometriosis is a complex set of symptoms linked to infertility, with well-studied clinical features and causes. However, it involves multiple factors and non-specific pathological mechanisms, including inflammation, cell invasion, angiogenesis, genetic mutations, steroid hormone metabolism, and oxidative stress [45,46]. Because its symptoms are nonspecific and it can be confused with other gynecological conditions like cancer, a more precise differential diagnosis is needed for endometriosis [47].

The development of non-invasive diagnostic methods is a key objective. This moment makes the diagnostic method easily available in any healthcare system. There are many imaging approaches to diagnose the endometriosis, such as transvaginal ultrasound examination and computed tomography. Additionally, there are nuclear medicine and X-ray, which are used a bit rarely [48]. The transvaginal ultrasound examination is widely used to diagnose endometriosis being much less invasive compared to laparoscopy [49,50], but it generally exhibits lower sensitivity and heavily depends on the expertise of the examining physician [48]. Although the current clinical classification of endometriosis is well established, the addition of minimally invasive laboratory tests based on peripheral blood biomarkers that reflect pathological changes in the body is of utmost importance. These tests can provide valuable information to complement existing diagnostic approaches [46,51].

There is a growing trend to avoid the invasive procedure of laparoscopy, even though it remains the “gold standard” for diagnosing endometriosis. Early, non-invasive diagnosis could significantly influence disease management and treatment options, whether medical or surgical [44,46,52]. The biomarker most consistently studied in endometriosis is CA 125. Nearly 30 years ago, Mol and colleagues observed that CA 125 might be more effective for diagnosing advanced stages (III–IV) than for earlier stages (I–II) [53]. However, CA 125, along with CA 43, has limited sensitivity and specificity. Proteins in venous blood, such as vascular endothelial growth factor (VEGF), urocortin, C-reactive protein (CRP), tumor necrosis factor alpha (TNF-alpha), interleukin-6 (IL-6), and follistatin, or protein sets, can offer certain diagnostic advantages. Nonetheless, their specificity remains low [17,54]. To overcome this, current approaches such as genomics, transcriptomics, proteomics, and metabolomics are used to identify more specific and sensitive biomarkers for endometriosis. Proteomics- and metabolomics-based studies are among the most commonly used. Evidence increasingly suggests that global metabolic profiles are valuable for identifying diagnostic biomarkers in easily accessible biofluids [51,55,56,57,58]. Metabolomics, in particular, have proven effective for monitoring disease progression [59] and distinguishing diseased from healthy states [60]. The primary aim of clinical metabolomics is to discover new biomarkers with high predictive value for practical medical use. Multiple metabolomics-based models for diagnosing endometriosis have been developed [50,55,56]. Promising metabolites include lipids [51,57], amino acids [18,58], and fatty acids [58]. It is important to balance the invasiveness of biological sampling methods with the predictive power of metabolites. Therefore, blood plasma and serum remain the most minimally invasive and informative sample types used in metabolomics studies.

Recent research highlights a notable difference in PUFAs levels between endometriosis patients and healthy women. Specifically, blood serum EPA levels tend to decrease in endometriosis [61], and consuming more EPA-rich foods can lower the disease risk and inflammatory response [27,28]. Additionally, improvements in pain symptoms have been linked to administration of EPA and other ω-3 fatty acids in these patients [59,60]. In our study, however, no significant differences in EPA and DHA levels were found between groups, consistent with other studies reporting no impact of dietary PUFAs [23,62]. Conversely, levels of ω-6-PUFAs, AA, and EDA increased in endometriosis patients, reflecting the disease’s inflammatory aspect. Still, earlier research did not show increased AA in endometriosis [61,63], and its intake did not directly raise disease risk [28]. This may be due to unexamined dietary habits among volunteers. The balance of fatty acids and lipids is a growing area of interest, with lipidomics-based metabolomics emerging as a promising method for diagnosing endometriosis [56,64,65]. Although not extensively studied in our research, lipidomics could be a logical next step based on our findings.

Beyond comparing PUFA levels, a quantitative enrichment analysis would be useful to identify the specific metabolic pathways which are altered in endometriosis. This method is a powerful method to evaluate the metabolic shifts [66]. Previous studies identified shifts toward aerobic glycolysis (the Warburg effect) and alterations in steroid metabolism in ectopic endometrial tissues [67,68]. These changes promote fatty acid oxidation [67], which we also observed, leading to a hypoxic microenvironment and mitochondrial dysfunction with increased reactive oxygen species [69]. In addition, molecules linked to inflammation, such as prostaglandins F2α and E2 [22], leukotrienes A4 and C4 [29], and thromboxane A2 [21], seem to be altered metabolic pathways. In previous assessments, the described PUFAs (AA, DHA, EDA, and EPA) significantly affected the pathways. The enrichment scores were 0.302 (*p* = 0.005) for arachidonic acid and 0.208 (*p* = 0.007) for linoleic/α-linolenic acid metabolism. However, our small metabolite set limits the reliability of these results. The small sample size also weakens statistical power, so we may miss truly significant pathways. We need to expand the metabolite panel to account for the impact. We also need to increase sample size to strengthen findings, reduce overfitting risk, and use multiple pathway databases and corrections.

The role of miR-451a in endometriosis development remains debated [70], but recent studies have highlighted its value for diagnosis, and it features in various diagnostic signatures [33,36,42,71]. Research focuses on miR-451a’s involvement in the macrophage migration inhibitory factor (Mif) pathway, which supports the survival of endometriosis lesions [72]. Our study also found a notable difference in salivary miR-451a levels, which were lower than in healthy controls, contrasting with other studies that reported increased serum levels of miR-451a [36,40]. Additionally, serum levels of miR-125b were previously shown to be significantly higher than those of other miRNAs in endometriosis [73,74]. However, Hajimaqsoudi et al. observed no significant difference between healthy controls and patients with eutopic endometriosis, noting only overexpression of miR-125b in the ectopic endometriosis group [75]. Since this group included only patients with adenomyosis, this may explain why salivary miR-125b levels were similar across groups. Overall, the role of miR-125b in endometriosis remains uncertain [76].

This prospective observational translational study aims to identify differentially expressed metabolites in serum samples from endometriosis patients using GC–MS-based metabolomics. The goal is to clarify the disease’s pathogenesis and discover new biomarkers for a non-invasive diagnostic test. Due to the complex sample preparation for GC–MS analysis and the individual and dietary variability of PUFAs, validating the bioanalytical method is essential for accurate diagnostics in the future [31,77]. The GC–MS method was developed and validated based on key parameters (accuracy, linearity, carryover, sensitivity, and specificity) in accordance with bioanalytical procedure guidelines. It proved to be sensitive (Table 2), linear (Table 3), and robust for measuring PUFAs in blood serum (Table 4). This routine method detects fatty acids in biological samples and costs approximately USD 40.00. Using the concentrations of AA and EDA in blood serum and miR-451a in saliva, a prognostic model for endometriosis was developed, demonstrating high sensitivity and specificity. Among the monitored parameters, EDA level is the most significant for distinguishing groups. EDA levels increased markedly in patients with endometriosis (*p* < 0.001), more than AA levels, and significantly contributed to predictive models across various ML approaches (Table 6, Figure 6). Although ω-6-PUFAs were not shown to influence endometriosis risk or severity significantly [23,63], our findings suggest a potential role in disease pathogenesis. The results also highlight the importance of a multiparametric approach to diagnosis, given the relatively low specificity of miRNAs and PUFAs compared to other gynecological diseases. Nonetheless, non-targeted metabolomics may identify new markers with greater predictive power [17], as demonstrated in our study. In our study, potential markers of endometriosis were identified among all the Folch-extracted and derivatized metabolites. Further evaluation of these markers is planned to develop a multiparametric model that is both informative and minimally parameterized, reducing costs and overfitting.

The developed model has certain limitations. Specifically, the sample sizes for both groups are small, making the results preliminary. Few volunteers specified their medications, and medication intake could not be controlled. Dietary habits were not comprehensively assessed, and they, along with comorbidities, might influence PUFA levels. Serum may not reflect PUFA composition as accurately as erythrocytes in whole blood; however, extracting PUFAs from serum is faster than the traditional alkaline hydrolysis of whole blood. It is also important to note that saliva is a variable biofluid, and its condition may depend on diet and oral hygiene, which were not fully examined. Further research using an independent patient cohort is necessary to validate this diagnostic model for endometriosis. The panel of low-molecular-weight metabolites and miRNAs was limited to four and two, respectively. Therefore, comprehensive omics-based methods that include a wider variety of metabolites and transcripts are still necessary to create the most effective model.

Future studies should expand this panel with additional promising metabolites, including those identified through untargeted metabolomics (Figure 2c), to enhance diagnostic accuracy. Given the future integration of metabolic and transcriptomic approaches [78], it should be emphasized that our study remains preliminary, with a limited set of metabolites, and that the integration remains model-based. The biological heterogeneity of endometriosis is an important factor that requires greater attention to develop comprehensive predictive and diagnostic models. Both metabolomic and transcriptomic approaches require batch-effect correction [79,80]. Although our model shows promising predictive features (Table 6), it was not validated on an independent dataset; it was built using 50% of all samples, with the other 50% for validation. Despite thorough cross-validation, this reduces the robustness of the results, and further refinement is necessary.

Overall, our pilot model combining serum PUFAs and salivary miR-451a demonstrates promising diagnostic potential and should be validated in an independent cohort. It aims to provide a more complete understanding of the biological changes in endometriosis by integrating phenotypic metabolic shifts with underlying genetic regulation. Using multiple biomarkers helps mitigate biases related to comorbidities or diet.

## 4. Materials and Methods

### 4.1. Patients and Study Design

This prospective observational study was carried out in the Clinical Hospital no. 123, Yu.M. Lopukhin Federal Research and Clinical Center of Physical-Chemical Medicine of Federal Medical Biological Agency. The trial was approved by the Institutional Ethics Committee of Yu. M. Lopukhin Federal Research and Clinical Center of Physical-Chemical Medicine of Federal Medical Biological Agency, Moscow, Russia (protocol no. 2024/10/24).

Fifty-two women aged between 28 and 44 (median age 39), who needed surgery for suspected endometriosis or other health issues, participated in the study. Exclusion criteria included recent hormonal treatment within two months, menopausal status, pregnancy, and pelvic inflammatory disease. Each participant signed a written informed consent form to ensure their anonymity. The study did not involve advertisements or financial compensation. Researchers collected clinical data, including socio-demographic information, symptoms, comorbidities, prior treatments, and ultrasound findings.

Alongside the transvaginal ultrasound, blood and saliva samples were collected from each patient after an overnight fast and transported to the laboratory within 1 h. Serum and saliva samples were stored at −80 °C for future analysis. Post-surgery, ultrasound reports were reviewed to confirm the diagnosis. Patients were then categorized into two groups: (1) 28 patients with symptoms and a transvaginal ultrasound diagnosis of endometriosis (endometriosis group, EM); (2) 24 asymptomatic patients or those with symptoms but no endometriosis diagnosis (healthy control group, HC). Patients with endometriosis had the following symptoms: dysmenorrhea, menorrhagia, chronic pelvic pain, and dyspareunia. Metabolomic and transcriptomic analysis was performed including these groups. Table 1 presents all demographic data. A total of 24 patients (15 with endometriosis, 9 without endometriosis) showed the presence of comorbidity; coronavirus infection, COVID-19 was in past history for 35 patients (17 with endometriosis, 18 without endometriosis); uterine surgery was made in 14 patients (10 with endometriosis, 4 without endometriosis); uterine fibroids were detected in 16 patients (12 with endometriosis, 4 without endometriosis); 15 patients (all belonging to the endometriosis group) had a surgical diagnosis of endometriosis.

Patients with endometriosis exhibited symptoms such as dysmenorrhea, menorrhagia, chronic pelvic pain, and dyspareunia. Metabolomic and transcriptomic analyses were conducted on these groups. Table 1 summarizes all demographic data. Among the patients, 24 (15 with endometriosis and 9 without) had comorbidities. A history of COVID-19 was noted in 35 patients (17 with endometriosis and 18 without). Uterine surgery was performed on 14 patients (10 with endometriosis and 4 without). Uterine fibroids were found in 16 patients (12 with endometriosis and 4 without). All 15 patients diagnosed surgically with endometriosis belonged to the endometriosis group.

### 4.2. Materials

Vacuette™ blood tubes containing coagulation activator for serum collection and Salivette™ tubes for saliva collection were purchased from Sarstedt AG (Nümbrecht, Germany). AA (purity ≥ 97.0%), DHA (purity ≥ 98.0%), EPA (purity ≥ 98.5%), and EDA (purity ≥ 98.0%) GC–MS analytical standards were purchased from Supelco (SigmaAldrich, St. Louis, MO, USA). Methanol (HPLC grade) was purchased from J.T. Baker (Amsterdam, The Netherlands). Chloroform (for analysis grade, purity ≥ 99.8%) and water (LC-MS grade) were purchased from Merck (Darmstadt, Germany). Hydrochloric acid (37.0%) was purchased from PanReac (Barcelona, Spain). Hexane, ethanol, and Na_2_CO_3_ powder were of analysis grade and purchased from Chimmed (Moscow, Russia). For RT-qPCR analysis, the ALMIR hsa-miR-451a-5p and ALMIR hsa-miR-125b-5p kits (Algimed Techno, Minsk, Belorussia) were used. Each kit contained standards for quantitative assay (8 levels), as well as ferment and reaction mixtures for reversal transcription and PCR. ExtractRNA mixture and nuclease-free water was purchased from Evrogen (Moscow, Russia). QuDye^®^ RNA BR kit was purchased from Lumiprobe, Ltd. (Moscow, Russia).

### 4.3. Polyusaturated Fatty Acids Extraction and Derivatization

The PUFAs were extracted using modified Folch method [81]. Briefly, Vacuette™ tubes with blood were centrifuged at 3000 rpm for 10 min. 40 μL of separated sera were transferred to an Eppendorf tube and mixed with 100 μL of methanol, and 200 μL of chloroform. Then, they were vortexed at 4000 rpm for 30 s and centrifuged at 14,000× *g* for 10 min at 4 °C. The hydrophobic phase was separated, and PUFAs contained in it were derivatized as follows: 150 μL of 8% HCl solution in methanol and 750 μL of methanol were added to 100 μL of the phase, mixed, and incubated at 80 °C for 1 h in EVA LC-S thermostatic evaporation system (VLM, Bielefeld, Germany). Then, 500 μL of hexane and 500 μL of 10% Na_2_CO_3_ solution in water were added, vortexed at 4000 rpm for 30 s and centrifuged at 14,000× *g* for 10 min at 4 C. The hexane layer was separated, and 1 μL was injected in chromatograph.

### 4.4. GC–MS Analysis

A microliter of the derivatized sample was injected in splitless mode into Kristall 5000.2 gas chromatograph (Chromatec, Yoshkar-Ola, Russia) coupled with single-quadrupole DSQ mass spectrometer (Thermo Finnigan, San Jose, CA, USA) equipped with a fused silica capillary column (30 m × 0.25 mm ID), chemically bound with 0.25 μM polar DB-23 phase (Agilent, Waltham, MA, USA). The carrier gas was helium (purity 99.9995%). The parameters were estimated as follows: injector temperature 250 °C, gas flow rate 1 mL/min, transfer line temperature 200 °C, ion source temperature 200 °C. The initial temperature of the column was kept at 50 °C for 2 min, then increased from 50 °C to 100 °C at 50 °C/min and to 230 °C at 10 °C/min and maintained at 230 °C for 5 min. Total run time was 14.2 min. The mass spectrometer was operated in the electron ionization (EI) mode at 70 eV. Chromatograms were acquired in full scan and single ion monitoring (SIM) modes. The full scan mass spectra were obtained at m/z range of 30–400 and scan rate 900 spectra/min, and used for untargeted metabolomic analysis and identification of targeted PUFAs (Figure S1a). The SIM mode was used for quantitative analysis of PUFAs (Figure S1b); target *m*/*z* were 79.1 (methyl esters of AA, EDA, EPA, and DHA) and 74.1 (for methyl ester of heneicosanoic acid, HEA, as internal standard) and dwell time was 50 ms. After instrumental analysis, the identification of the metabolites was carried out using the standard NIST mass spectra library (v.2.0, 2001) and when available, by comparison with authentic standards. Data were processed using xCalibur v.1.4 software; peak detection and deconvolution were performed. Concentrations of discriminant metabolites were obtained using the internal standard method by the area ratio.

The GC–MS method was evaluated for linearity, range, LOD, and LOQ. Validation of the analysis method was performed according to [82]. For the determination of linearity, LOD and precision, both within batch and day-to-day, different concentrations of FAMEs (0.005, 0.05, 0.25, 0.5, 2.5, 5, 10, 25, 50 and 250 μg/mL) were prepared in hexane with a final concentration of 25 μL/mL of HEA as internal standard.

### 4.5. RNA Sample Extraction and Preparation

Saliva was collected in Salivette™ tubes by the patients under the control of the medical staff and centrifuged immediately to collect followed by storage at −80 °C for the analysis of miRNAs. Total RNA was extracted from ~250 μL of saliva using the ExtraRNA reagent according to the manufacturer’s specifications followed by extraction by phenol and chloroform. Briefly, 250 µL of saliva was mixed together with 750 µL of ExtraRNA reagent, incubated at room temperature followed by followed by centrifugation at 4 °C and 13,200× *g* for 10 min. A total of 200 µL of chloroform was added to the supernatant and mixed well, followed by centrifugation at 4 °C and 13,200× *g* for 15 min. After centrifugation, the supernatant (about 400 µL) was transferred to a new Eppendorf tube with added 400 µL of isopropyl alcohol and mixed well. The sample was then placed on ice and incubated for 10 min. After that, the samples were centrifuged at 13,200× *g* and 4 °C for 15 min, and the supernatant was discarded. An amount of 1 mL of 80% ethanol was then added and the precipitate was fully resuspended through repeated pipetting, centrifuged (4 °C, 13,200× *g*, 15 min) followed by discard of supernatant. The procedure was performed twice, and the precipitate containing total RNA dried at room temperature. Total RNA was solved with 200 μL of nuclease-free water. The yield of RNA was assessed using Fluo 200 fluorimeter (Allsheng, Hangzhou, China) using the QuDye^®^ RNA BR kit according to manufacturer’s protocol. Briefly, 20 μL of sample RNA solution was added to 180 μL of fluorescent dye working solution (1 μL of concentrated QuDye^®^ dye per 198 μL of TE-buffer solution), mixed for several seconds, centrifuged for dropping, and incubated for 2 min at room temperature in the dark. The fluorescence of the mixture was measured at laser λ_ex_ 630/λ_em_ 660 nm. The concentration of RNA was quantified by the calibration curve.

### 4.6. RT-qPCR

Total RNA (~25 ng) from each sample was reverse transcribed using ALMIR hsa-miR-451a-5p and ALMIR hsa-miR-125b-5p kits following the manufacturer’s instructions. Levels of miRNAs were quantified by RT-qPCR using the calibrants from the kits with the specific forward primers miR-125b and miR-451a and the universal reverse primer complementary to the anchor primer. The thermal cycling conditions were initiated by denaturation at 95 °C for 15 min, followed by 45 cycles at 95 °C for 5 s and annealing at 60 °C for 15 s. Threshold cycle and melting curves were acquired by using the quantitation and melting curve program of the Bio-Rad Manager (Bio-Rad Laboratories, Hercules, CA, USA). The relative miRNA levels were determined using the comparative crossing point (*C_p_*) method by the calibration curve.

### 4.7. Statistical Analysis

Statistical analysis was performed using scripts in the R language [83] in the RStudio (version 2024.12.0) environment [84] with “randomForest”, “caret”, “pROC” packages (Supplementary File S1 contains scripts). The data for analysis were normalized by median, mean-centered, and auto-centered using “MetaboAnalystR” package (version 3.3.0) for R [85]. To prevent the data leakage, all the parameters (PUFAs concentrations, miRNAs amount, and the approved diagnosis) to build the model were obtained, and the same parameters were used to test the model. The molecules for classification logistic models were selected by their variable importance in projection (VIP) greater than 1 according to the orthogonal projection on latent structures discriminant analysis (OPLS-DA) with “ropls” package (version 1.18.0) for R [86]. The selected molecules were used in logistic regression models. The response variable was the group diagnosis. The no endometriosis case was assigned a value of “0” and endometriosis case was assigned a value of “1”. The final classification model was built based on 20 logistic regressions. The final diagnosis was determined based on which of them received the most “votes”. Each intermediate model was tested by 20-fold cross-validation to determine its sensitivity, specificity, and threshold. The accuracy of each diagnosis was assessed as the ratio of the number of true cases of a diagnosis to the total number of cases with such a diagnosis given by the model. The results of ROC-analysis were visualized with “ggplot2” package (version 3.5.1).

Untargeted metabolomics raw files were transformed in mzML format, using MSConvert GUI 3.0.23170-df762d9 (ProteoWizard software, v. 3.0.23170) [87] followed by processing using “xcms” (version 1.58.0) [88], “MsExperiment” (version 1.12.0) [89], and “pheatmap” (version 1.0.12) packages. TICs data were normalized for inter-group comparison.

## 5. Conclusions

This study identified significant differences in the polyunsaturated fatty acid composition of blood serum and the miR-125b/miR-451a profile in saliva, supporting their potential as diagnostic markers for endometriosis. The biomarker-based model showed good sensitivity and specificity for diagnosis. Combining metabolomic and transcriptomic methods could lead to the discovery of new molecular markers, improving non-invasive diagnosis and risk assessment. These advances are expected to improve diagnostic accuracy and facilitate personalized treatment options for endometriosis.

## Figures and Tables

**Table 1:** Demographic data of the enrolled patients.

Variables	Endometriosis (*n* = 28)	Healthy (*n* = 24)	*p* Value
Age (median, range)	39 (24–45)	34 (24–44)	0.001
Body Mass index (median, range)	24.0 (19.3–40.4)	23.5 (18.3–36.3)	
Ethnicity			
Caucasian	28	24	–
Age at menarche, mean	12.9	13.4	0.321
Cigarette smoking%	17.9	29.2	0.335
**Pain syndrome**			
Chronic pelvic pain%	82.1	45.8	0.006
Pain intensity%			
*Mild*	52.2	88.9	0.033
*Moderate*	39.1	11.0	0.124
*Severe*	8.7	0.0	0.361
Dyspareunia%	87.5	59.3	0.024
Dysmenorrhea%	23.1	4.2	0.054
Menorrhagia%	57.1	12.5	<0.001
Prolonged menstruations%	17.9	4.2	0.123
Vaginal spotting before/after menstruations%	64.3	12.5	<0.001
Menstrual cycle < 21 days (median, range)	10.7	12.5	0.841
Cycle day	18 (2–27)	14.5 (0–34)	0.387
**Parity**			
Normal%	71.4	41.7	0.056
Caesarean%	21.4	16.7	0.727
In-time%	78.6	45.8	0.031
Premature births%	3.6	0.0	0.354
Abortions%	35.7	20.8	0.367
Miscarriages%	42.9	16.7	0.082
Complications after abortion	36.4	16.7	0.378
Pregnancy complications%	25.0	20.8	0.689
Myoma%	42.9	16.7	0.051
Surgical interventions on uterus%	35.7	16.7	0.145
Hyperplasia of endometrium%	57.1	16.7	0.003
**Discomfort**			
Headache%	32.1	8.3	0.036
Vertigo%	25.0	16.7	0.422
Nervous state%	64.3	45.8	0.182
Bad mood%	46.4	45.8	0.966
Performance decrement%	39.3	16.7	0.073
Sleeping disorders%	14.3	4.2	0.217
**Comorbidities**			
Vegetovascular dystonia%	35.7	12.5	0.054
Depression%	25.0	12.5	0.254
Gynaecological inflammatory disorders%	28.6	25.0	0.772
Psychotherapy%	7.1	0.0	0.182
Thyroid disorders%	14.3	4.2	0.235
Anaemia%	53.6	37.5	0.197
Gastrointestinal disorders%	53.6	25.0	0.036
Urinoexcretory disorders%	35.7	25.0	0.404
COVID-19%	60.7	75.0	0.274
Cardiovascular disorders%	25.0	16.7	0.422

**Table 2:** Linearity estimation.

Compound	Range (μg/mL)	Linearity (*r*^2^)	Slope	Intercept	Limit of Detection (μg/mL)	Limit of Quantitation (μg/mL)
*Cis*, *cis*-11,14-Eicosadienoic acid, EDA	3.0–125.0	0.9954	0.0026	0.0030	1.08	3.23
Arachidonic (*all-cis*-eicosatetraenoic) acid, AA	2.0–125.0	0.9973	0.0014	−0.0052	0.52	2.00
*all-cis*-Eicosapentaenoic acid, EPA	1.0–125.0	0.9915	0.0066	−0.0197	0.27	1.00
*all-cis*-Docosahexaenoic acid, DHA	7.5–250.0	0.9929	0.0033	−0.0953	2.37	7.50

**Table 3:** Estimation of electivity and carry-over for the determination of FAMEs.

Compound	Selectivity (%of LOQ Peak Area)	Carry-Over (%of LOQ Peak Area)
EDA	7.2%	14.8%
AA	0.0%	7.8%
EPA	0.0%	1.5%
DHA	0.0%	7.7%
HEA	0.0%	0.0%

**Table 4:** Within- and between-run accuracy and precision for the determination of FAMEs.

Compound	LLOQ		QC Low		QC Medium		QC High	
	Accuracy (%)	Precision (RSD%)	Accuracy (%)	Precision (RSD%)	Accuracy (%)	Precision (RSD%)	Accuracy (%)	Precision (RSD%)
Within-run (*n* = 5)								
EDA	94.9	5.13	97.4	2.6	94.7	5.3	93.4	6.6
AA	97.9	2.1	89.9	10.1	107.4	7.4	104.1	8.9
EPA	100.9	0.9	91.6	8.4	95.6	4.4	95.6	4.3
DHA	95.0	5.0	96.1	3.9	96.9	3.1	86.5	13.5
Between-run (*n* = 3)								
EDA	105.5	5.5	102.7	2.7	92.7	7.3	91.0	9.0
AA	93.7	6.1	90.9	9.1	108.4	8.4	102.6	2.6
EPA	96.1	3.9	91.3	1.8	95.7	4.3	95.4	4.6
DHA	87.7	12.3	100.2	4.5	94.3	5.7	89.6	10.4

**Table 5:** Contingency table for differential diagnosis of endometriosis. Values in parentheses indicate the accuracy of each diagnosis.

Group	Healthy Control	Endometriosis	Total	Percentage of Corrected
OPLS-DA				
Healthy control	24	0	24	100.0%
Endometriosis	4	24	28	83.3%
Total	28	24	52	91.6%
SVM				
Healthy control	23	1	24	95.8%
Endometriosis	1	27	28	96.4%
Total	24	28	52	96.1%
Random forest				
Healthy control	22	2	24	91.7%
Endometriosis	3	25	28	89.3%
Total	24	27	52	90.4%

**Table 6:** Diagnostic performance (AUC, sensitivity, and specificity) and variables of the logistic regression model for endometriosis and healthy volunteers. Coefficient β, standard error of mean SE β, Wald criteria Z, and coefficient zero-probability *p* are provided.

Group	Sensitivity	Specificity	AUC (CI AUC)	Variables	β	SE β	Z	*p*
Endometriosis vs. Healthy control	0.958	0.964	0.994 (0.976–1.000)	Intercept	−1.405	0.758	−2.432	0.014
				EDA	1.130	0.489	3.484	0.010
				AA	0.008	0.005	2.510	0.048
				miR-451a	2.808 × 10^−7^	2.0 × 10^−7^	1.386	0.044

## Data Availability

The original contributions presented in this study are included in the article/Supplementary Materials. Further inquiries can be directed to the corresponding author.

## Supplementary Materials

The following supporting information can be downloaded at: https://www.mdpi.com/article/10.3390/ijms27073052/s1.

## Abbreviations

The following abbreviations are used in this manuscript:

AA	Arachidonic (*all-cis*-eicosatetraenoic) acid
DHA	*all-cis*-Docosahexaenoic acid
EDA	*Cis*, *cis*-11,14-Eicosadienoic acid
EPA	*all-cis*-Eicosapentaenoic acid
HEA	Heneicosanoic acid
GC–MS	Gas chromatography coupled with mass-spectrometric detection
LOQ	Lower limit of quantitation
LOD	Limit of detection
ML	Machine learning
OPLS-DA	Discriminant analysis with orthogonal projection on latent structures
PUFAs	Polyunsaturated fatty acids
ROC	Receiver operation characteristic
RT-qPCR	Quantitative reverse transcription polymerase chain reaction
SVM	Support vector machine learning

## Appendix A

**Table A1:** Contingency tables for differential diagnosis of endometriosis. The data for training sample and test sample are presented.

Group	Healthy Control	Endometriosis	Total	Percentage of Corrected
Training sample				
Healthy control	11	1	12	91.7%
Endometriosis	0	14	14	100.0%
Total	11	15	26	95.8%
Test sample				
Healthy control	10	2	12	83.3%
Endometriosis	0	14	14	100.0%
Total	10	16	26	91.7%
