# Supplementary material for: Integrating Metabolic and MicroRNA Profiling to the Diagnostics of Endometriosis: A Pilot Study
Source: Int J Mol Sci. 2026 Mar 27;27(7):3052. doi: 10.3390/ijms27073052 (PMC13073272; doi:10.3390/ijms27073052)
Supplement: Supplementary file 1 [file ijms-27-03052-s001.zip › ijms-4168063-Supplementary Figure S1.pdf]

International Journal of
Molecular Sciences

MDPI

Figure S1: (a), The total ion chromatogram view for the sample extracted from blood serum of the patient with endometriosis (red) and healthy volunteer (blue). (b), Selected ion chromatogram (m/z 79.1, quantifier) for the same samples.

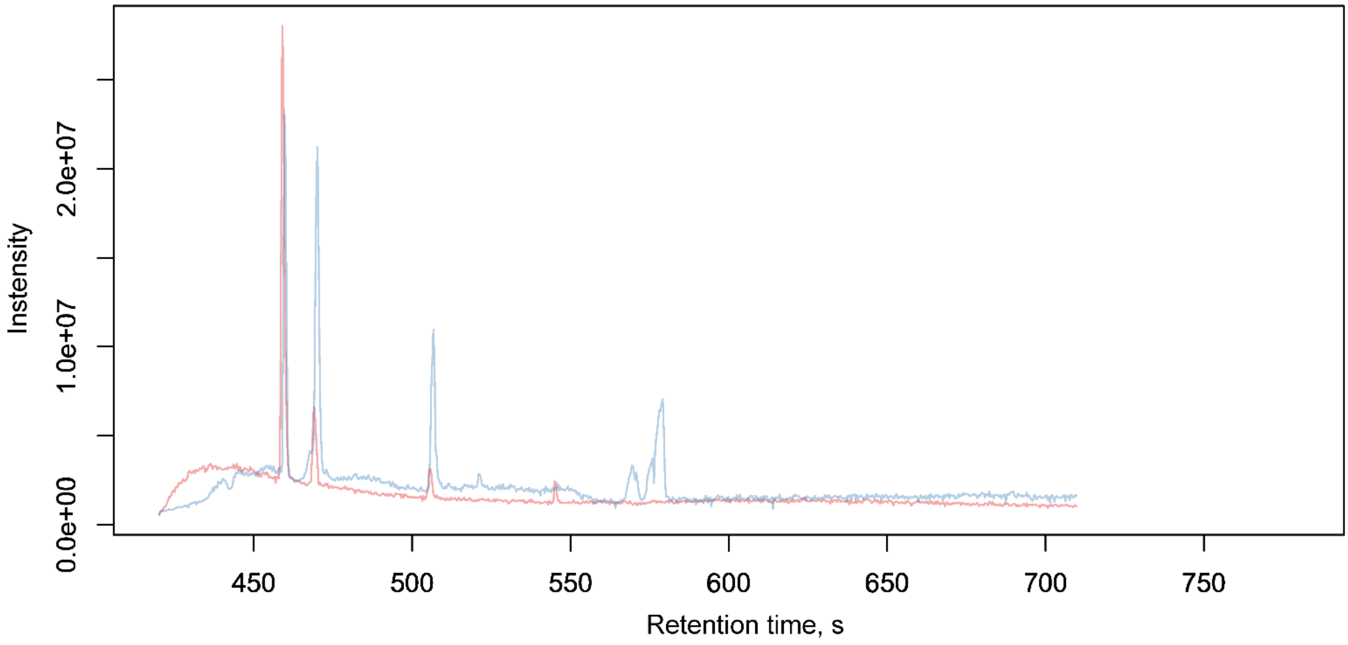

a

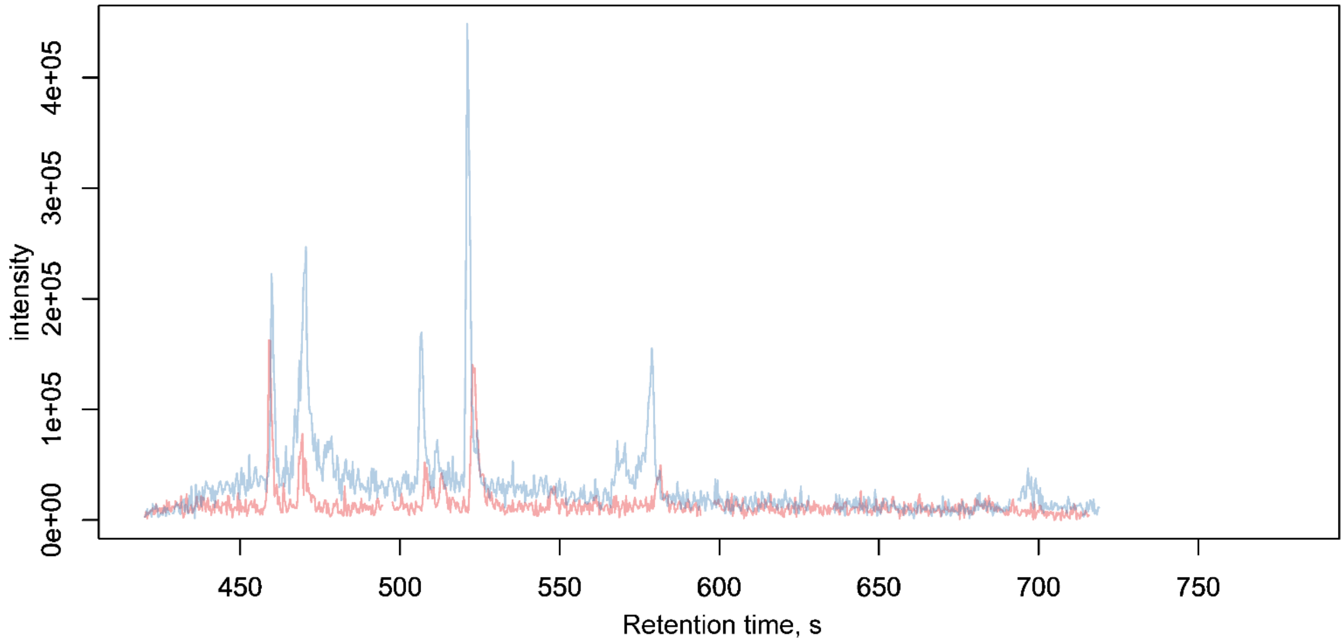

b
