# Supplementary material for: Integrating Metabolic and MicroRNA Profiling to the Diagnostics of Endometriosis: A Pilot Study
Source: Int J Mol Sci. 2026 Mar 27;27(7):3052. doi: 10.3390/ijms27073052 (PMC13073272; doi:10.3390/ijms27073052)
Supplement: Supplementary file 1 [file ijms-27-03052-s001.zip › ijms-4168063-Supplementary File S1.pdf]

```
#Loading libraries-------
library(readr)
library(readxl)
library(readxl)
library(dplyr)
library(tidyverse)
library(GGally)

#Data fecthing---------------------------------------------------------------
data <- read_excel(filename.xlsx, sheet = “data”)
data <- na.omit(data)
data <- as.data.frame(data)
names <- as.data.frame(data$Sample)
rownames(data) <- data[,1]
data[,1] <- NULL
data$Group <- as.factor(data$Group)
data <- na.omit(data)
mat <- data[,-Group]
data <- as.data.frame(data)

#OPLS-DA---------------------------------------------------------------------------------
library(ropls)
data.oplsda <- opls(data[,-Group], data$Group, predI = 2, orthoI = 1, crossvalI = 5, subset = "odd")
plot(data.oplsda, typeVc = "x-score")

#VIP metric
gr <- data$Group
#train <- data.oplsda@subsetVi
table(gr[data.oplsda@subsetVi], fitted(data.oplsda))
table(gr[-data.oplsda@subsetVi], predict(data.oplsda))
table(gr[train], fitted(data.oplsda))
table(gr[-data.oplsda@subsetVi], predict(data.oplsda, data[-data.oplsda@subsetVi]))
```

```
#correlations---------------------------------------------------------------------------------
library(corrplot)
library(ggcorrplot)
col<- colorRampPalette(c( "navy",  "darkgrey", "darkorange"))(20)
# mat : is a matrix of data
# ... : further arguments to pass to the native R cor.test function
cor.mtest <- function(mat, ...) {
  mat <- as.matrix(mat)
  n <- ncol(mat)
  p.mat<- matrix(NA, n, n)
  diag(p.mat) <- 0
  for (i in 1:(n - 1)) {
    for (j in (i + 1):n) {
      tmp <- cor.test(mat[, i], mat[, j], ...)
      p.mat[i, j] <- p.mat[j, i] <- tmp$p.value
    }
  }
  colnames(p.mat) <- rownames(p.mat) <- colnames(mat)
  p.mat
}
# matrix of the p-value of the correlation
p.mat <- cor.mtest(mat)
head(p.mat[, 1:6])
corrplot(cor(mat), method="circle", type="upper", order="hclust", col = col,
         number.cex = 0.1,  p.mat = p.mat, sig.level = 0.05)

#Heatmaps---------------------------------------------------------------------------------
library(pheatmap)
pheatmap(data[,-Group])
my_row <- data.frame(Group = rep(c("hc", "EM"), c(24,28)))
row.names(my_row) <- rownames(data)
```

```
pheatmap(data[,-Group], scale = "column", show_rownames = TRUE, angle_col = 0, annotation_row =
my_row)


#Boxplots ----------------------------------------------------------
library(ggrepel)
library(ggforce)
library(ggplot2)
library(ggpubr)
library(ggsci)
library(ggsignif)
library(cowplot)
# Box plot facetted by PUFAs
boxplot <- read_excel(filename.xlsx, sheet = “PUFAs”)
acids <- ggboxplot(boxplot, x = "Group", y = "Concentration", add = "jitter",
               color = "Group", palette = c("navy", "darkorange", "darkgrey"),
               facet.by = "Compound", short.panel.labs = TRUE,
               legend = "top",  ylab = " Concentration, ug/mL")
acids1 <- acids + facet_wrap(vars(Compound), scales = "free_y") +
  scale_y_continuous(expand = expansion(mult = 0.1)) +
  theme(axis.text.x = element_blank(), axis.ticks.x = element_blank())+
  stat_compare_means(method = "anova", label = "p.format", label.x = 1.75)
acids1


#ROC-analysis---------------------------------------------------------------------------------
library(glm2)
library(pROC)
library(cutpointr)
data <- read_excel(filename.xlsx, sheet = "ROC_data")
data <- as.data.frame(data)
rownames(data) <- data[,1]
data[,1] <- NULL
data <- data[,-Group]
model <- glm(Group ~ ., data = data, family = binomial)
```

```
rocobj <- plot.roc(data$Group, model$fitted.values,  colorize = FALSE,  type="l",lwd=3, col="#0f4060", ylim = c(0,1),
        xlim = c(1,0), legacy.axes=TRUE, xlab="1-Specificity", ylab="Sensitivity", print.auc=TRUE,
        ci = TRUE,print.auc.y=0.52, print.thres=TRUE)
ciobj <- ci.se(rocobj,                        # CI of sensitivity
             specificities = seq(0, 1, 0.05))
plot(ciobj, type = "shape", col = "#1c61b6AA")

cp <- cutpointr(x = model$fitted.values, class = data$Group)
cp[,2:8]

#LDA-------------------------------------------------------------------------
library(caret)
library(MASS)
data <- read_excel(filename.xlsx, sheet = "data")
data <- na.omit(data)
data <- as.data.frame(data)
rownames(data) <- data[,1]
data[,1] <- NULL
data$Group <- as.factor(data$Group)
mat <- data[,-1]
data <- as.data.frame(data)
f <- data[,-Group]
f$Group <- data$Group

set.seed(123)
training.samples <- f$Group %>%
  createDataPartition(p = 0.75, list = FALSE)
train.data <- f[training.samples, ]
test.data <- f[-training.samples, ]
prepparam <- train.data %>%
  preProcess(method = c("center", "scale"))
train.transformed <- prepparam %>% predict(train.data)
```

```
test.transformed <- prepparam %>% predict(test.data)

model <- lda(Group~., data = train.transformed)

predictions <- model %>% predict(test.transformed)

mean(predictions$class==test.transformed$Group)

plot(model)

head(predictions$class, 6)

head(predictions$posterior, 6)

head(predictions$x, 3)

lda.data <- cbind(train.transformed, predict(model)$x)


#SVM---------

set.seed(123)

training.samples <- data$Group %>%

  createDataPartition(p = 0.8, list = FALSE)

train.data  <- data[training.samples, ]

test.data <- data[-training.samples, ]


model <- train(

  Group ~ ., data = data, method = "svmLinear",

  trControl = trainControl("cv", number = 100),

  preProcess = c("center","scale")

)

predicted.classes <- model %>% predict(test.data)

head(predicted.classes)

mean(predicted.classes == test.data$Group)


set.seed(123)

model <- train(

  Group ~., data = train.data, method = "svmLinear",

  trControl = trainControl("cv", number = 100),

  tuneGrid = expand.grid(C = seq(0, 2, length = 100)),

  preProcess = c("center","scale")
```

```
)
plot(model)


#Random forest-------------------
library(randomForest)
set.seed(123)
training.samples <- data$Group %>%
  createDataPartition(p = 0.75, list = FALSE)
train.data  <- data[training.samples, ]
test.data <- data[-training.samples, ]


model <- randomForest(Group ~ ., data = data, ntree = 500,
                      mtry = sqrt(3), proximity=TRUE, importance = TRUE)
predictions <- predict(model, newdata = test.data)
table(test.data$Group, predictions)
importance(model)
varImpPlot(model)


## Lollipop plot
ggplot(varImpPlot(model), aes(x=reorder(rownames(model$importance), MeanDecreaseGini), y=MeanDecreaseGini,
)) + geom_point(aes(colour = MeanDecreaseGini), size = 5) +
  scale_color_gradient(low = "#0f4060", high = "#ed693b") +

geom_segment(aes(x=rownames(model$importance),xend=rownames(model$importance),y=0,yend=MeanDecreaseGini-0.2),  lineend = "square") +
  ylab("MeanDecreaseGini") +
  xlab("Parameter") +
  coord_flip() + theme_minimal()
```
